# Development of a Community-Based Integrated Service Model of Health and Social Care for Older Adults Living Alone

**DOI:** 10.3390/ijerph18020825

**Published:** 2021-01-19

**Authors:** Yu Mi Yi, Yeon-Hwan Park, BeLong Cho, Kyung-Choon Lim, Soong-Nang Jang, Sun Ju Chang, Hana Ko, Eun-Young Noh, So Im Ryu

**Affiliations:** 1 College of Nursing, Kyungnam College of Information and Technology, Busan 47011, Korea; yym7493@snu.ac.kr; 2 College of Nursing, Seoul National University, Seoul 03080, Korea; changsj@snu.ac.kr; 3 The Research Institute of Nursing Science, Seoul National University, Seoul 03080, Korea; nossje@snu.ac.kr (E.-Y.N.); soimaym@snu.ac.kr (S.I.R.); 4 Department of Family Medicine, College of Medicine, Seoul National University, Seoul 03080, Korea; belong@snu.ac.kr; 5 College of Nursing, Sungshin University, Seoul 02844, Korea; kclim@sungshin.ac.kr; 6 Red Cross College of Nursing, Chung-Ang University, Seoul 06974, Korea; sjang@cau.ac.kr; 7 College of Nursing, Gachon University, 191 Hambakmoero, Yeonsu-gu, Incheon 21936, Korea; Hanago11@gachon.ac.kr

**Keywords:** integrated service, community-dwelling older adults, healthcare, daily life support

## Abstract

The number of elderly people living alone worldwide is increasing, and the responsibility of the state in this context is emerging. This study aimed to develop a community-based integrated service (CBIS) model of health and social care for older adults living alone. The model was designed based on a literature review of previous community care models and per older adults’ health and daily life needs. Thereafter, feedback on the integrated model was taken from older adults living alone by conducting a survey (*n* = 1023) and focus group interviews, after which the opinions of the Public type Health Management Promotion Council were considered and content validity was confirmed. The model, comprising eight healthcare services and five social care services, was tested on 22 older adults for two weeks to assess its feasibility and preliminary efficiency. Each service included screening, assessment, providing service, evaluation, and quit. Participants rated their overall satisfaction with the services as 9 out of 10. Care navigators reported feeling comforted and discovered their own sense of being while providing the services. We believe that the CBIS model may foster independence among community-dwelling older adults living alone, thereby improving their quality of life through “aging in place”.

## 1. Introduction

Population aging has led to various social problems and affected various types of social services, healthcare, policies, and benefits that older adults use [1,2]. Additionally, changes to the family structure, such as those in family values, the growing prevalence of nuclear families, and the movement of the younger generation to big cities, have been detrimental to the amount of traditional family support that older adults receive.

As the population ages and the number of older adults living alone increases, societies throughout the world face the responsibility to build new support systems and provide novel forms of care for independence and happiness of sick or frail older people [3]. The important goal of welfare policies for older people is to provide appropriate services [4] that allow older adults to stay in their homes and communities instead of entering institutional care. Gerontological studies suggest that older adults typically wish to stay in familiar spaces like their homes, and by doing so, they can maintain their well-being [5]. Consequently, policies to introduce “aging in place” are being considered around the world [5,6,7], and various efforts have been made to introduce community care in Korea [8,9,10,11,12].

Older people may have illnesses, disabilities, or dysfunctionality that makes them dependent on daily care. Further, older people who live alone are more likely to report emotional problems [13,14] and are at a higher risk for early mortality [15] than those who live with others. Thus, older people living alone may require home care and specialized services such as community outings, meal delivery, financial support, housework support, and so on, as well as healthcare services for mental and physical support [16]. Optimizing social services and healthcare for older adults living alone can help them maintain their independence and promote their physical and mental health [17]. Several initiatives have attempted to enhance the integration of care in several countries [6,18,19]. However, these integrated care services are mainly at the provincial level, and individuals who need services that are more diverse can end up facing higher costs for insurance and care [18]. Through the National Health Insurance and Long-term Elderly Care Insurance System, the South Korean government has implemented healthcare and daily life support services for older adults [20]. Therefore, a supply system has been established to provide health and daily life support services for older adults. In South Korea, various community care programs for older adults living alone are being implemented at different levels such as at the national, local, and private level, including the Comprehensive Senior Support Center Program, the Community Care Program, and the Visiting Healthcare Program. However, these welfare services have limitations such as low accessibility, owing to diversification of suppliers, service redundancies, and a lack of a medical supply services [4,21].

Therefore, we developed a community-based integrated service (CBIS) model by improving and integrating the existing services and utilizing local resources, to establish an aged-friendly integrated service system so that older adults living alone can live comfortably and securely in the community and can continue to participate in society. We conducted a pilot test to gauge the preliminary feasibility of the CBIS model and to modify and supplement effectiveness measurement variables. The pilot test reviewed feasibility and sustainability by considering recipients’ satisfaction levels, fidelity, engagement, and acceptability.

## 2. Materials and Methods

### 2.1. Study Design

Our development process included service needs based on targeted literature reviews and study findings from a preliminary survey and focus group interviews with older adults living alone, followed by CBIS model drafts based on opinions of the Public type Health Management Promotion Council and the reviews of five experts. Thereafter, we implemented a pilot test to 22 older adults for two weeks. Analyses were performed to evaluate feasibility according to program fidelity and participants’ satisfaction levels with each service. For fidelity, five researchers conducted randomized service observations and informed the care navigators to keep a detailed activity diary each time they provided a service. Participants were asked to rate their satisfaction level with the service they received on a 10-point scale (1 = strong dissatisfaction, 10 = strong satisfaction). The service satisfaction level questionnaire included items regarding the adequacy of the content, time required, immediacy, convenience, kindness, and overall satisfaction level with the Health and Daily Life Support (HDLS) Center. Service sustainability was verified through engagement and acceptability. For engagement, we reviewed the care navigator activity records and conducted a group interview of the care navigators. Acceptability was confirmed through the possibility of job maintenance for older adults, a local human resource, by calculating the weekly working hours of the care navigators. This was a newly developed intervention; therefore, a control group was not utilized.

### 2.2. Development of the CBIS Model

**Figure 1:**
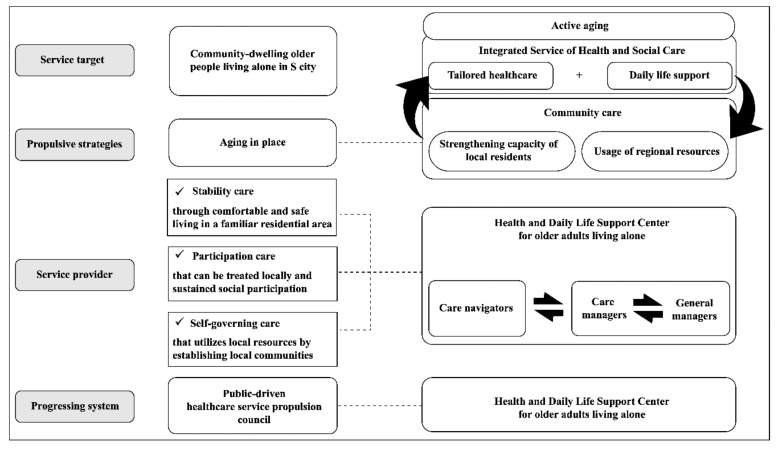
Overview of the community-based integrated service model.

A multidisciplinary team formed a public private academic council with public officials and community stakeholders (the Public type Health Management Promotion Council) in Siheung city and prepared a draft for the CBIS model through regular quarterly meetings and occasional meetings with working groups and reflected the opinions of the local community. Integrated domestic and international service models and delivery systems for community healthcare and welfare [6,7,8,9,10,11,12,18,19,22,23] were reviewed to consider connecting links and differentiation from existing programs, and the existing services and delivery systems were enhanced and included in the CBIS model. Key concepts of the CBIS model were adopted from key tenets of the Korean government’s public healthcare and social care projects: resource linkage in the Community Care Program, case management in the Visiting Healthcare Program, and service types and practice methods in the Comprehensive Senior Support Center Program for older adults living alone. Three key elements of the service model were basic daily life support, tailored healthcare and community primary medical system connectivity module, and the conjugation of local resources. The CBIS included the following modules: First, the daily life support module comprised self-help support that enabled older adults to live independently, support concerning various hazards within their residential environment, and an integrated call center to provide instrumental support as needed. Second, the tailored healthcare module, a service area that had been overlooked, comprised emotional support to foster psychological stability and quality of life, cognitive enhancement programs to prevent the inflow of a long-term care facility for dementia, and supervised medication administration, chronic disease management, and linkage support for the local primary medical system or medical/health products to properly treat health problems and prevent diseases. Finally, the local resource utilization module consisted of continuous and integrated inclusion of local businesses and local older adults to strengthen local residents’ capabilities. Figure 1 shows an overview of the CBIS model.

**Figure 2:**
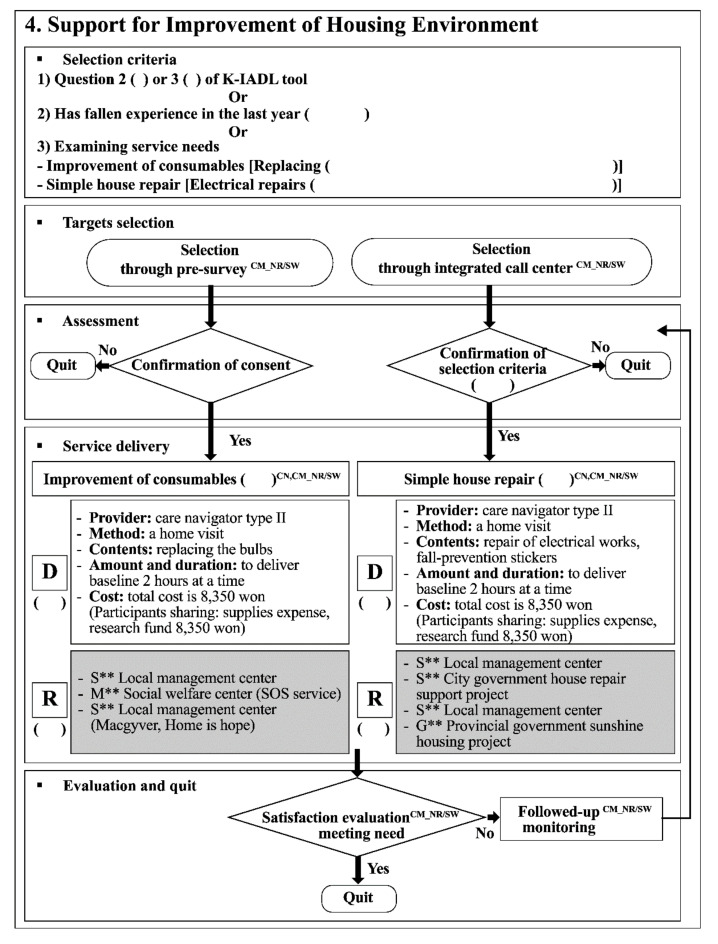
Example of an algorithm. K-IADL: Korean instrumental activities of daily living scale; GM: general manager; CM: care manager; NR: nurse; SW: social worker; CN: care navigator; D: direct service provider; R: reference to existing service; Won: unit of Korean currency, 8350 won is about 7 U.S. dollars.

Feedback was received through the first survey [24] and focus group interviews [25]. This original study was conducted to identify essential service needs and delivery methods for enhancing service accessibility. We identified the community service needs from our survey results [24] (Supplemental Figure S1). In total, 52.6% (*n* = 538) of participants were depressed and 25.1% (*n* = 258) experienced falls. Participants with mild cognitive impairment and dementia were 26.7% (*n* = 273) and 30.5% (*n* = 312), respectively. Each participant had an average of 5.23 diseases and were taking an average of 3.5 prescription drugs per day. Although 49.3% (*n* = 504) of the participants were currently receiving services provided by public institutions, it was reported that there were service needs such as meal preparation 23.8% (*n* = 243), meals on wheels 29.6% (*n* = 303), housing repairs 59.7% (*n* = 611), outing support 16.5% (*n* = 169), medical/health product linkage 58.9% (*n* = 603), and assessment of older adults’ general condition over the phone 60.3% (*n* = 617). Both women (*n* = 796) and men (*n* = 227) had a high need for safety and healthcare services. The demand for meals was high for men, but the need for meals on wheels was similar for men and women [24]. These health conditions and essential service needs were reflected in the content of the model. Therefore, the CBIS model comprised eight healthcare services, five social care services, and tailored case management. The eight healthcare services were: (1) cognitive enhancement program, (2) self-management for chronic illness, (3) supervised medication administration corresponding to healthcare support, (4) emotional support, (5) emergency support, (6) medical/health product linkage support that identifies and provides information on whether medical/health products can be purchased or rented, (7) community primary medical system linkages through regular medical care and medical checkups for older patients with chronic diseases using local primary medical institutions, and (8) accompaniment to the hospital. The five social care services were: (1) meal support, including cooking services and meals on wheels, (2) cleaning/washing, (3) delivery of ingredients corresponding to housework support, (4) improving the residential environment, through replacement of consumables, fall prevention stickers, and simple housing repairs, and (5) community outings. Focus group interviews were conducted among 58 older adults living alone, in eight groups of 7–8 people each. The focus group interviews identified three themes of service experiences and needs among older adults living alone: (1) the lack of information about service availability and accessibility methods, (2) the pros and cons of service experiences, such as experiencing positive emotions through communication with visitors and the burden of time management when a provider visited for a house-based service respectively, and (3) the need for client-oriented services to provide services when they are actually required [25]. These findings were reflected in delivery methods for enhancing service accessibility. In order to unify the service delivery systems and to identify and evaluate changes in service needs and conditions of consumers—and not simply integrate health and daily life services—a HDLS Center was established in the public health center. An integrated call center that can be accessed at any time when service is needed was installed in the HDLS Center. The service delivery process was organized into five steps: screening, assessment, service provision, evaluation, and quit (Supplemental Figure S2). In order to visualize the CBIS model and standardize the implementation procedures, a service manual for the CBIS model and a one-page practical algorithm of the service (Figure 2) were developed.

The service manual for the CBIS model included an overview, a HDLS Center, a service target, service content, and service delivery personnel. We held a public hearing of the Public type Health Management Promotion Council to gather opinions from the community. Then, we sought input from a panel of experts/scholars to validate the service manual of the CBIS model. The panel consisted of five professors in social welfare and nursing. The mean of items in the service manual was 4.62, ranging from 4.2 to 4.8 (item range 1–5) and the individual content validity indices (CVIs) were excellent, with scores higher than 0.78 for all items except for item “service rationale.” Although one item score was lower than 0.78, the experts suggested that the underlying construct for this service rationale was clear and that it would be better to clarify the specificity of the older people living alone in the research area so that the acceptability of local users can be increased. The scale CVI was high (CVI = 0.87) (Table 1). The survey feedback, as well as the opinions of the experts and the promotion council, guided the additional revisions of the intervention protocol and service manual.

### 2.3. Pilot Testing of CBIS

#### 2.3.1. Participants

Potential participants for this pilot study included 1057 older people living alone who participated in the first (*n* = 796) and second (*n* = 261) surveys. In South Korea, the percentage of older adults living alone was 23.6% in 2017 [26]. This study was conducted after a randomized selection of 50 out of 177 participants from D-district (of all districts, this had the largest number of older people living alone). Participants were deemed eligible to participate if they lived alone and met service needs or per-service delivery criteria in the first and second baseline assessments and provided informed consent. After excluding potential participants for various reasons, 22 participants were finally recruited for the pilot study (Supplemental Figure S3) and received at least one service. We notified participants about the program over e-mail and made reminder telephone calls. Participants were not compensated other than receiving the services provided. During the pilot test, daily life support and healthcare services were provided 21 times and 29 times, respectively.

#### 2.3.2. Procedure

The pilot study included a service criteria assessment, a two-week intervention, and two follow-up measures (after each service and full intervention). In this pilot study, the daily life support module included meal preparation (*n* = 0), meals on wheels (*n* = 8), grocery delivery (*n* = 0), cleaning/washing (*n* = 5), community outings (*n* = 3), fall prevention stickers (*n* = 4), and simple home repairs (*n* = 1). The tailored healthcare module included providing a conversational partner (visit + phone; *n* = 4), assessing older adults’ general condition over the phone (*n* = 9), accompanying them to the hospital (*n* = 0), medical/health product linkage (*n* = 0), supervised medication administration (*n* = 9), and playing cognitive function-enhancing games (*n* = 7). Each participant was provided with more than one of the 13 services.

As part of the community strategy, we utilized the “Local Management Center” (an institution for revitalizing housing welfare such as simple house repairs) and the existing meals on wheels business, “Chan and Bob,” and hired and trained healthy older adults in the area as care navigators.

Service delivery personnel were subdivided into general managers, care managers, care navigators, and care-navigator managers. General managers continuously managed the overall operation status and educated the care managers, the care-navigator managers, and the care navigators. Care managers managed targets, service delivery, and linked community resources. Care navigators provided direct services, which were divided into three types: (1) daily life support services, (2) meals on wheels and housing repairs, and (3) assisting with the cognitive function-enhancing games and confirming participants’ general condition over the phone. Care-navigator managers were responsible for the overall management of the recruitment and placement of care navigators while communicating with the general managers. A general manager and care managers were stationed at the HDLS Center in the public health center, which manages the inflow of new targets including receiving emergency calls. Six researchers—five nursing doctoral students and one social worker with extensive training in service process and intervention development—served as intervention facilitators for all groups as general managers. They strictly adhered to the standard written intervention protocol for each service. They educated five care navigators and one care-navigator manager.

#### 2.3.3. Ethics Statement

This study was reviewed and approved by the institutional review board of Seoul National University Hospital (IRB NO. 1809-131-961). Participants were informed that the information provided would be kept confidential and anonymous and that they were free to withdraw from the study at any time. The study was conducted after obtaining written consent.

## 3. Results

### 3.1. Participant’s Characteristics

Of the 22 individuals who participated in the pilot test, most were women (86%). Participants’ mean age was 80.04 (±6.18) years (range = 70–89 years). The pilot test participants had an average of 8.09 service requests in the survey asking for service needs, but an average of 2.27 services received through the actual pilot study (Supplemental Figure S4). The main reasons that the participants refused the services that they wanted to receive in the pre-survey in the actual pilot test were as follows: “I can do it alone,” “I do not need it yet,” and “I do not want others to visit my house.” Participants preferred attaching fall prevention stickers among the housing welfare services. During the pilot study, 13 services were provided, and four types of services were not provided: meal preparation, grocery delivery, coordination of access to medical products and services, and accompanying to the hospital. The most frequently used services were supervised medication administration (*n* = 9) and confirming general condition over the phone (*n* = 9), followed by the cognitive function-enhancing game (*n* = 7).

### 3.2. Service Feasibility

Concerning fidelity, five researchers conducted randomized service observations and asked the care navigators to keep a detailed activity diary each time they provided a service. During the pilot test period, we used daily worksheets, weekly team meetings, frequent phone contact, and daily discussions of each schedule to maintain fidelity to the intervention service.

As demonstrated in Table 2, the overall service satisfaction level was high (around 9 out of 10). Participants were most satisfied with the medication administration service (9.5) and the fall prevention stickers (9.6), and they were least satisfied with the care providers checking on their general condition over the phone (7.6) and outing support (8.4). Participants who received healthcare services rather than daily life support services, except for housing repairs, were generally more satisfied with the HDLS Center. For most services, participants were satisfied with the providers’ kindness; however, they rated the adequacy of the content and convenience as low, especially for community outings and assessing their general condition by phone.

### 3.3. Service Sustainability

Five care navigators provided a total of 89.5 h of service during the two-week pilot test: One care navigator provided an average of 17.9 h of service in the two weeks. The average times spent as a conversation partner on the phone and confirming older adults’ general condition over the phone were similar.

A group interview was conducted with the care navigators immediately after the pilot test to identify the care navigators’ experience and problems when providing services. Most care navigators were highly positive about the experience of having provided services for older adults living alone. They described feeling comforted and discovered their own sense of being during the process of providing the services. Regarding their experiences in the service delivery process, care navigators described a lack of prior information about service users (participants’ sex, precautions, illnesses) and countermeasures in the event of an accident, difficulties in forming relationships when providing services over the phone and communication with the care navigator manager such as delayed service schedule notifications, and sudden change in service schedule.

## 4. Discussion

We developed and pilot-tested a service model to provide customized healthcare and daily life support to older people living alone through a tailored and integrated community-based service model that adopted age-friendly principles. The key elements of our CBIS model were providing basic daily life support, tailored healthcare and community primary care, and support seeking local resources; therefore, the model may ultimately help community-dwelling older adults’ “aging in place” [17].

Healthcare services were higher in needs and utilization than daily life support services. Among healthcare services, users were also highly satisfied with health and emotional support service provisions such as supervised medication administration and having a conversational partner by phone or visiting. This can be explained by the fact that the main issues for older adults living alone are economic difficulties (40.5%), health problems (25.0%), and loneliness (22.7%) [16]. Participants’ satisfaction with and demand for supervised medication administration can be explained by the fact that taking medicine is a complex process for older people living alone, given that it requires both cognitive and physical capabilities [27]. Living alone poses a risk of isolation, loneliness, depression, and suicidal ideation [13,14]. Since it is difficult to openly reveal these negative emotions, having a conversational partner may be effective in helping older adults form ties and express emotions [28]. Conversation partners act as supporters [28], and the readily accessible private conversations may provide comfort and stability. In addition, general managers were able to enhance users’ satisfaction with the service by reflecting personal preferences, such as preparing and coordinating a space for conversations in the HDLS Center for people who wanted a conversational partner but were reluctant to converse at their own home.

In contrast, confirmation of general condition by phone, one form of emotional support, and the cognitive function-enhancing games were high service needs but recorded the least satisfaction level. Although having a conversation partner over the phone and confirmation of general condition by phone were separate emotional support services, care navigators expressed difficulties in applying these two services in different ways and in forming relationships with the participant only by phone. Since older adults who prefer phone services persevere on their own and tend to believe that they have to deal with their own problems independently, they may not reveal their problems [29], and services provided only over the phone have limitations concerning forming bonds and expressing negative emotions [30].

Every service user wants to receive the service in a location that is convenient psychologically, informationally, and geographically, whenever he/she wants, using simple procedures [4]. A cognitive function-enhancing game service, in which participants completed cognitive tasks, provided immediate feedback, and recorded the results continuously, limited accessibility because many participants were unfamiliar with using a computer and needed help, and only one machine was installed in the HDLS Center. Older adults typically fear memory loss associated with aging and developing dementia, and it is more difficult for those living alone to detect cognitive impairment or dysfunction than for those living with others [31]. Therefore, service providers should enhance accessibility and recognize all targets as potential service consumers—not just older people living alone who directly express their service needs—and ensure continuous monitoring of cognitive functions and connecting in-need people to appropriate support when necessary.

In addition, participants who received healthcare were highly satisfied with the HDLS Center. These findings tentatively suggest that the integration of healthcare and daily life support may be effective in enhancing users’ overall satisfaction with the service system. Therefore, social service models for older adults must consider health services.

Actual usage of daily life support services was low, followed by meals on wheels, cleaning/washing, fall prevention stickers, outing support, and housing repairs. Housing repairs, fall prevention stickers, and cleaning/washing services were highly satisfactory, while meals on wheels and outing support services were less satisfactory. Despite having the highest needs, the housing repairs service, which provided only one actual instance of consumption, showed the highest level of satisfaction, but future research is needed to confirm this result. The fall prevention stickers were highly satisfactory and the most preferred service among housing welfare services, as older adults who live alone fear falling more and have a greater fall history than those living with others [14,26]. Since fall prevention and reducing the fear of falling are both main objectives of gerontological care [14], continuous fall-preventive interventions should be implemented through activities to check and improve the home environment that can cause falls.

Further, participants who received cleaning/washing services rated their overall satisfaction with these individual services highly; however, their overall satisfaction with the HDLS Center was low. This could reflect a lack of service differentiation, as daily life support services such as cleaning/washing services have been provided through various welfare service systems in the past.

Older people living alone are very vulnerable regarding their caloric intake, nutrition, and eating habits due to a lack of meal diversity and difficulties associated with purchasing groceries [32]. Participants in this pilot study sought the meals on wheels service, reported that they were reluctant to let others visit their home, and preferred delivery services as their health and cognitive functions declined [28,29], but their service satisfaction level was low. Various strategies, including quantitative and qualitative improvements in nutrition and habitual changes through nutrition education, were needed to improve meal support services. Older people with physical disabilities wanted independent outing [26], so the outing support service had the lowest level of satisfaction.

When providing a service, it is necessary to pay attention not only to users’ satisfaction but also service providers’ satisfaction [30]. The utilization of community resources and healthy older adults in the recruitment and training of care navigators was useful in promoting psychological intimacy between users and providers. Maintenance of continuous service and service accessibility may improve service effectiveness and efficiency [4]. In principle, public-type work demands at least 7–8 h a week, and social service-type work, 15 h a week [26]. Local stakeholders involved in the senior employment program insist that at least 9 h of work per week should be guaranteed to ensure business continuity. This pilot test met this requirement and confirmed the sustainability of service delivery using community resources. In this respect, CBIS, which allows older adults to participate in jobs that offer care for older people, may result in positive social and economic effects and increase the life satisfaction [33] of older adults in the community.

Securing professional personnel is a prerequisite for better service [4,21], given that care providers should serve as a vital source of interaction. Service hours, content, and methods vary depending on the care navigator, and differences in these activities can affect the target population [30]. Consequently, a systematic and detailed job-specific service practice manual should be provided to clarify the work of the care providers and to secure professional personnel.

Standardized service delivery and reporting systems are needed to ensure consistent service delivery by establishing an effective and efficient communication system at the HDLS Center. Efficient communication is required to minimize errors in service delivery by systematically managing and transmitting information, such as providing prior information on the sex and characteristics of the user or reporting an emergency situation before the service is provided.

During the pilot test, there was no consumption of coordination of access to medical products and services or accompaniment to the hospital. We think this is because these two services are conditional once-off services, rather than services required at all times. These results may reflect our limited sample size and the short intervention duration. Additionally, the fact that this was a pilot test with a small sample size limits the applicability of the noted services. This study is a pilot test to confirm feasibility before a large pilot project, and future studies should be conducted by employing a larger sample size, a longer intervention period, and more resources. If a larger sample is assessed, the most needed services can be identified, and so can their association with users’ personal characteristics, which may help in creating tailor-made services for older adults living alone.

## 5. Conclusions

This study provides preliminary evidence that CBIS is a feasible augmentation to tailored and integrated services provided for older adults who live alone. Despite the noted limitations, the pilot test showed the possibility of addressing older adults’ daily lives and health service needs, as well as establishing and applying an age-friendly service system using community resources that utilizes older adults as a human resource. Preliminary evidence suggests that the CBIS model, designed to integrate daily life support and healthcare, may help older people living alone maintain their independence and help realize “aging in place”—an effort to address the diverse needs and aspirations of older adults—an international and national imperative by employing age-friendly practical principles. Future research is needed to clarify the use of tailored CBIS in the community.

## Figures and Tables

**Table 1:** Item means and individual content validity index (CVI) for the Community-Based Integrated Service (CBIS) model service manual.

Service Manual ITEMS		Mean ± SD	Individual CVIs
Service overview	Service purpose	4.6 ± 0.5	0.85
	Service rationale	4.8 ± 0.4	0.75
HDLS Center	HDLS Center management	4.8 ± 0.4	0.9
	HDLS Center guideline	4.8 ± 0.4	0.9
	HDLS Center administration	4.4 ± 0.5	0.85
service target	selection criteria	4.2 ± 0.8	0.85
	selection procedures	4.6 ± 0.5	0.9
	target management	4.6 ± 0.5	0.9
	Quit management	4.6 ± 0.5	0.85
service content	Algorithms	4.6 ± 0.5	0.9
	detailed contents	4.4 ± 0.5	0.9
service delivery personnel	General manager task	4.6 ± 0.5	0.95
	Care manager task	4.6 ± 0.5	0.8
	care navigator task	4.8 ± 0.4	0.85
	Others (co-operative physician, care-navigator managers)	4.6 ± 0.5	0.85
		Total means 4.62	Scale CVI 0.87

**Table 2:** Service satisfaction level and service delivery times.

Service Type		Service Satisfaction Level																		Service Delivery Times		
		Adequacy of Content			Time			Immediacy			Convenience			Kindness			Overall Satisfactionwith Center			Minutes		
		Sex			Sex			Sex			Sex			Sex			Sex					
		W	M	T	W	M	T	W	M	T	W	M	T	W	M	T	W	M	T	Mean	Min.	Max.
S2		9.3	8.3	9.1	8.8	8	8.6	9	7.5	8.6	8.6	8	8.4	9.6	9.5	9.6	9.1	8.5	8.9	8.8	7	10
S3		9.3	10	9.4	9.3	10	9.4	9	10	9.1	8.9	10	9	9.6	10	9.6	8.4	10	8.6	125	90	180
S5		10	-	10	10	-	10	10	-	10	10	-	10	10	-	10	10	-	10	15	15	15
S6		8.8	-	8.8	9.8	-		9.8	-	9.8	9.8	-	9.8	9.8	-	9.8	8.8	-	8.8	30	30	60
S7		7.5	-	7.5	8	-	8	9	-	9	7.5	-	7.5	10	-	10	9	-	9	70	60	90
S10		7.7	8.3	7.8	7.5	7.7	7.5	6.5	7.5	7.2	7.5	7.5	7.5	8.3	7.7	8.2	8.7	8	8.6	3.9	1.2	18.4
S11	V	9.5	8.3	9.2	9.5	8.3	9.2	8.9	8.7	8.8	9.4	8.3	9.1	9.5	8	9.1	9.3	8.7	9.1	50	30	60
	P																			5.3	1.1	10
S12		8.4	-	8.4	8	-	8	8.6	-	8.6	8.5	-	8.5	9.2	-	9.2	9	-	9	38.7	26	58
S13		9.4	5	9.1	9.8	7	9.6	9.8	5	9.5	9.8	5	9.5	9.8	7	9.6	10	6	9.7	60	60	60
Total		8.9	8.0	8.8	9	8.2	8.9	9	7.7	9.0	8.9	7.8	8.8	9.5	8.4	9.5	9.1	8.2	9			

S2, Service 2: meals on wheels, S3, Service 3: cleaning/washing, S5, Service 5: housing repairs, S6, Service 6: fall-prevention sticker, S7, Service 7: community outings, S10, Service 10: confirmation of general condition by phone, S11, Service 11: conversational partner (V, visit; P, phone), S12, Service 12: cognitive function-enhancing game, S13, Service 13: supervised medication administration, M, men; W, women; T, total.

## Data Availability

Not applicable.

## Supplementary Materials

The following are available online at https://www.mdpi.com/1660-4601/18/2/825/s1. Figure S1: Community service needs (first survey). Figure S2: Service delivery in the community-based integrated service model. Figure S3: Flow of participants through the study. Figure S4: Services provided in the pilot test per participants’ sex.
