# Supplementary material for: Development of a Community-Based Integrated Service Model of Health and Social Care for Older Adults Living Alone
Source: Int J Environ Res Public Health. 2021 Jan 19;18(2):825. doi: 10.3390/ijerph18020825 (PMC7835935; doi:10.3390/ijerph18020825)
Supplement: Supplementary file 1 [file ijerph-18-00825-s001.pdf]

Supplemental Figures

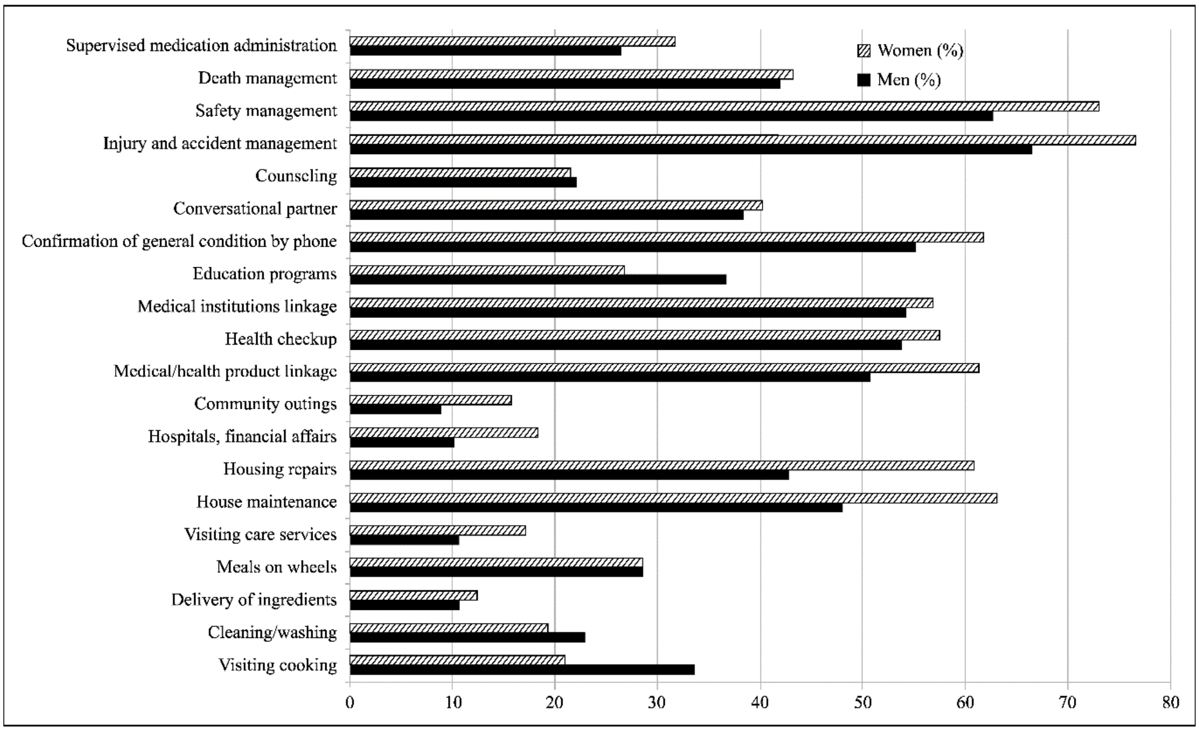

Supplemental Figure S1. Community service needs (first survey).

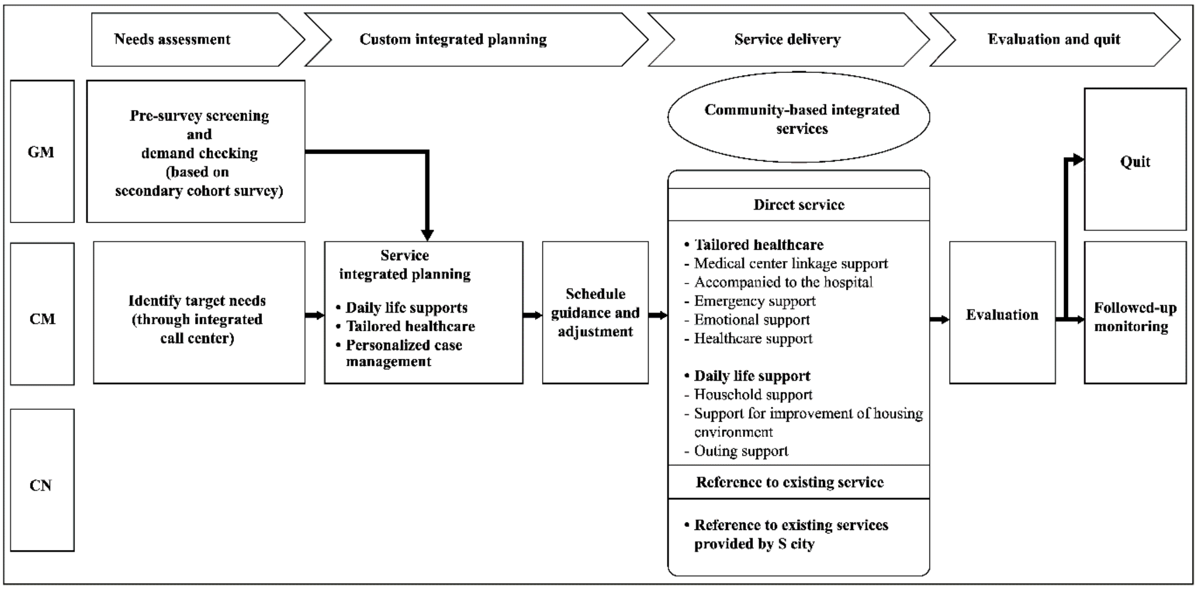

Supplemental Figure S2. Service delivery in the community-based integrated service model.

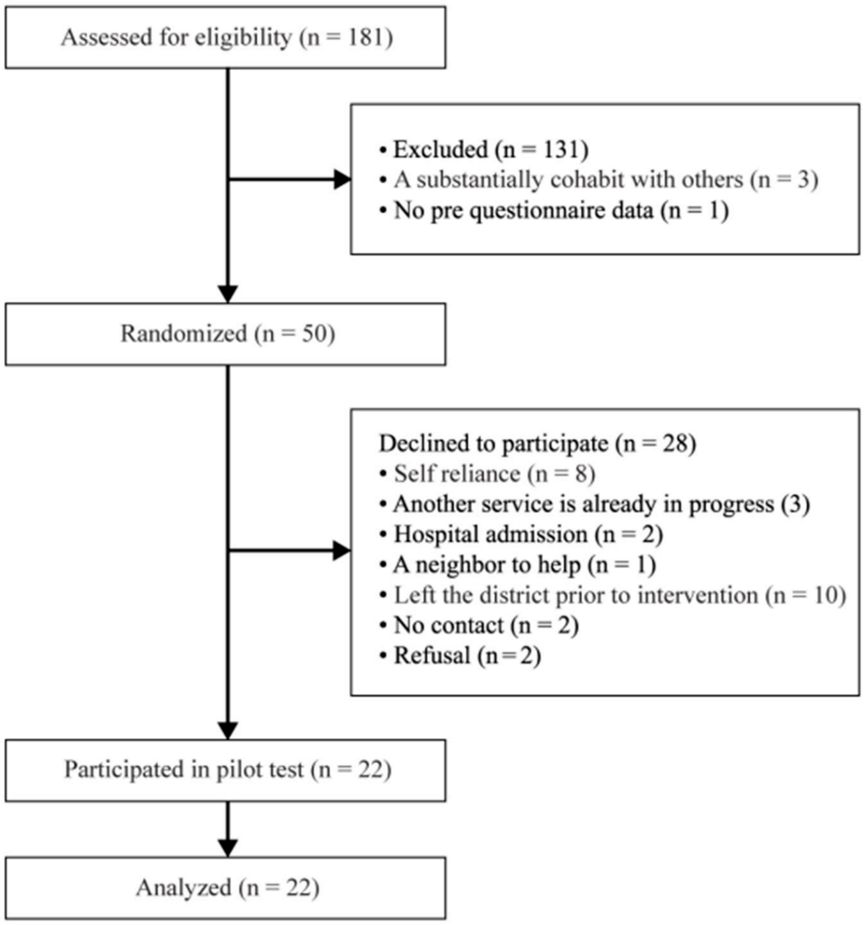

Supplemental Figure S3. Flow of participants through the study.

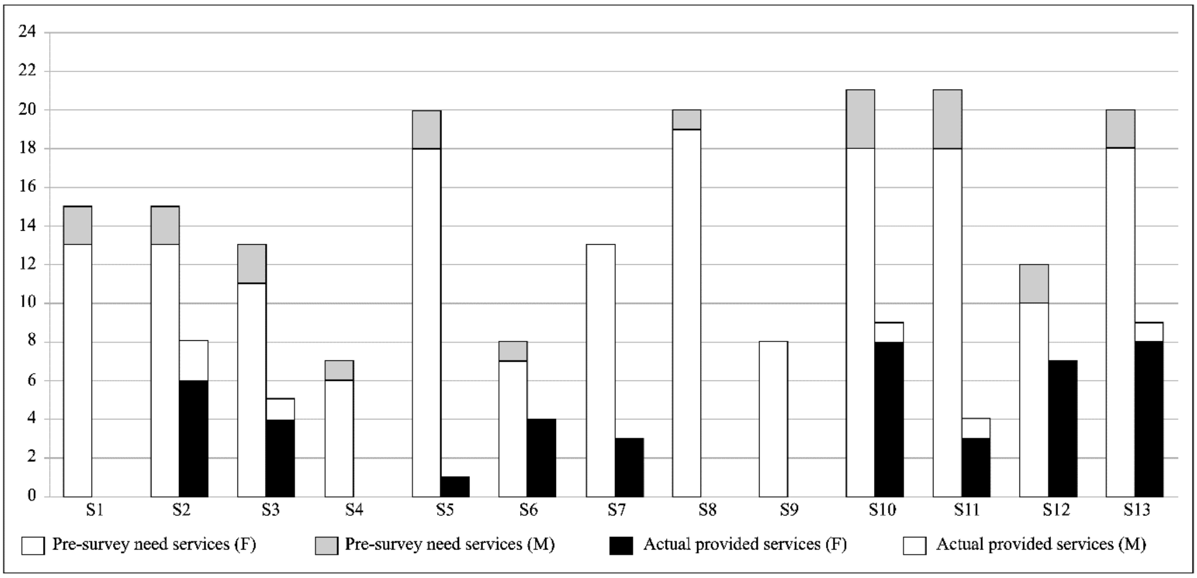

Supplemental Figure S4. Services provided in the pilot test per participants' sex. S1: meal preparation; S2: meals on wheels; S3: cleaning/washing; S4: delivery of groceries; S5: housing repairs; S6: fall prevention stickers; S7: community outings; S8: coordination of access to medical products and services; S9: accompanying to the hospital; S10: confirmation of general condition by phone; S11: conversational partner (visit + phone); S12: cognitive function-enhancing game; S13: supervised medication administration.
